# Long Non-Coding RNA DUXAP10 Promotes Tumorigenesis and Metastasis in Anaplastic Thyroid Cancer

**DOI:** 10.3390/cancers17233852

**Published:** 2025-11-30

**Authors:** Nicole R. DeSouza, Michelle Carnazza, Tara Jarboe, Danielle Quaranto, Kaci Kopec, Anthony J. Centone, Kate Nielsen, Robert Suriano, Augustine Moscatello, Humayun K. Islam, Xiu-Min Li, Jan Geliebter, Raj K. Tiwari

**Affiliations:** 1 Department of Pathology, Microbiology & Immunology, New York Medical College, Valhalla, NY 10595, USAtseymour@student.touro.edu (T.J.); humayun.islam@wmchealth.org (H.K.I.); xiumin_li@nymc.edu (X.-M.L.); raj_tiwari@nymc.edu (R.K.T.); 2 School of Natural and Mathematical Sciences, University of Mount Saint Vincent, Riverdale, NY 10471, USA; 3 Department of Otolaryngology, Westchester Medical Center, Valhalla, NY 10595, USA; 4 Department of Pathology, Westchester Medical Center, Valhalla, NY 10595, USA

**Keywords:** long non-coding RNAs, miRNAs, regulatory non-coding variants, ribodiagnostics, RNA therapeutics, targeted therapy, cancer

## Abstract

**Simple Summary:**

Anaplastic thyroid cancer is a fast-growing and deadly malignancy. A major reason for its extremely low survival rate is the absence of effective diagnostic, prognostic, and therapeutic tools that have significantly improved outcomes in other cancer types. A long non-coding RNA molecule investigation presents a novel and unique approach for combating anaplastic thyroid cancer and may contribute to finding the answers needed to control and treat this disease. Identifying dysregulated long non-coding RNAs may reveal previously unknown molecular mechanisms of ATC and provide potential diagnostic, prognostic, or therapeutic targets. These molecules often display regulatory capabilities that extend beyond those of proteins or other protein-coding genes. The ability of long non-coding RNAs to fine-tune the genome and control phenotypic output makes them extremely attractive avenues of study and investigation. We found that reducing the expression of a single long non-coding RNA, DUXAP10, significantly diminished multiple cancer-associated phenotypes of anaplastic thyroid cancer. DUXAP10 expression is significantly higher in some patients with anaplastic thyroid cancer, and we developed a cell model that describes the consequences of the anaplastic thyroid cancer proliferative and metastatic cascade when this RNA molecule is overexpressed, and how these cancer-promoting mechanisms can be alleviated by gene-specific targeting and lowering of this RNA molecule’s expression.

**Abstract:**

**Background**: Long non-coding RNAs (lncRNAs) are regulatory molecules that have multifaceted impacts on the carcinogenic molecular landscape—with pathologic consequences when aberrantly expressed. Anaplastic thyroid cancer (ATC) is a rapidly progressing and highly lethal malignancy, with mortality rates approaching 100%. The molecular/transcriptomic signature of ATC has significant gaps in understanding; thus, a comprehensive study of ATC non-coding RNA transcript regulation is necessary. **Results**: The lncRNA Double Homeobox A Pseudogene 10 (DUXAP10) was identified in patient genomic datasets as a highly upregulated transcript in ATC vs. normal thyroid tissue. DUXAP10 expression was transcriptionally repressed with CRISPR-interference (CRISPRi), and data supports an extensive role of DUXAP10 in several cancer-promoting phenotypes in ATC, both in vitro and in vivo. Our two DUXAP10-CRISPRi cell lines significantly reduced the rapid growth and metastatic behaviors characteristic of ATC, affecting proliferation, viability, clonogenicity, apoptosis, invasion, migration, tumorigenesis, and metastasis. **Conclusion**: Thus, DUXAP10 is a proposed prognostic marker and therapeutic target for ATC disease propagation and progression.

## 1. Introduction

Anaplastic Thyroid Cancer (ATC) is one of the most lethal and fast-growing metastatic malignancies [1]. A key challenge in ATC research is that it arises from many of the same driver mutations (e.g., *BRAF^V600E^*) found in papillary thyroid cancer (PTC), a slow-growing and highly treatable carcinoma [2]. Both ATC and PTC arise from thyroid follicular cells; however, the rapid progression of ATC is supported by its loss of follicular cell differentiation and acquisition of a high mutational burden—an oncogenic process not typically exhibited by PTC [3]. Therefore, mutational studies alone are insufficient because ATC and PTC share similar cellular origins and many of the same mutations [4]. The use of small molecule inhibitor therapy to treat PTC is successful in its curative efforts, whereas in ATC, these treatment modalities are relatively futile [5]. Further, there are significant hurdles to be overcome when diagnosing and treating ATC—challenges that are not present in PTC. The identification of biomarkers that can contribute to early detection or serve as a molecular target to aid in therapeutic successes is pertinent.

Emerging evidence suggests that the aggressive and metastatic nature of ATC is driven, in part, by aberrant epigenetic mechanisms [6]. The identification of differentially expressed genes (DEGs) that arise and drive feed-forward disease progression surpasses the forthcomings of mutational study [7,8]. The consideration that this aggressive carcinoma is driven by wild-type DEGs allows for a full-range molecular analysis of ATC’s unique cellular processes [8]. A central focus of epigenetic processes in cancer has led to the discovery of long non-coding RNA (lncRNA) molecules [9]. These molecules have extensive DNA-, RNA-, and protein-binding domains that enable both broad and fine-tuned impacts on the transformation of cancer cells and the orchestration of cancer phenotypes [10,11].

The differential expression patterns of lncRNAs have enabled the elucidation of unknown molecular mechanisms that drive the various hallmarks of cancer (i.e., immortality, invasive potential, migratory propensity, clonogenic capacity, etc.) [12]. Using publicly available National Center for Biotechnology Information (NCBI) Gene Expression Omnibus (GEO) datasets, we aimed to identify differentially expressed lncRNAs for further molecular evaluation. Double Homeobox A Pseudogene 10 (DUXAP10) was the top statistically significant differentially expressed gene in ATC compared to normal thyroid tissue (GSE33630) and has been previously established as an overexpressed oncogene in various carcinomas [13]. However, its role in ATC has not yet been explored. DUXAP10, a large lncRNA molecule, has been described for its ability to regulate a broad spectrum of cellular processes, making this molecule indispensable for evaluation. In the present study, we aimed to examine the effects of its overexpression on ATC’s phenotypic landscape, and we report a novel regulatory role in ATC.

## 2. Materials and Methods

### 2.1. Cell Lines

ATC cell line T238 (RRID:CVCL_6299) and immortalized normal thyroid follicular epithelial cell line Nthy-ori-3-1 (RRID:CVCL_2659) were maintained in complete media composed of 10% FBS 1X RPMI 1640 + L-glutamine and phenol red pH indicator. Two genetically engineered cell lines, T238-ΔDUXAP10#1 and T238-ΔDUXAP10#2, were generated using lentiviral transduction and confirmed with qRT-PCR and RNAseq analysis. HEK293T cells (CRL-3216) used for lentiviral harvesting were maintained in complete media composed of 10% FBS 1X DMEM + 4.5 g/L glucose, L-glutamine, and sodium pyruvate, and phenol red pH indicator. Cells were grown in a cell culture water-jacketed incubator at 37 °C and 5% CO_2_. Cell passage was completed using a 0.25% Trypsin, 2.21 mM EDTA, and 1X [-] sodium bicarbonate solution. Processes for cell maintenance and passage were completed as previously described [14].

### 2.2. Bioinformatic Analysis

Publicly available NCBI GEO datasets were used for selection of a DEG using the provided GEO2R software. *Limma* package and GEO2R query combined R version 2.11.0 and Bioconductor 2.6 platforms for identification and ranking of the top 250 DEGs in ATC vs. normal thyroid tissue (GSE33630). RNAseq data provided by NCBI GEO was analyzed using the DESeq2 R package to generate normalized transcripts per million (TPM) expression counts (GSE85457).

### 2.3. qRT-PCR

Total RNA was extracted from 175 cm^2^ tissue culture flasks at 70% confluence using Zymo Research Quick RNA Miniprep Kit (cat no. 11237) according to manufacturer’s protocol. RNA concentrations and sample purity was determined using an IMPLEN Nanophotometer Pearl (serial no. 3464) nanodrop reader. RNA with a concentration of 50 ng/μL were used for qRT-PCR.

Primer sequences of approximately 18–30 bases in length were designed using NCBI Blast and ordered dry from GeneWiz. Primer set for DUXAP10 was F: 5′-GGTTCAACAGTATGGCTCCAAAG-3′; R: 5′-GACTGCCATCCACAGATGAAG-3′. GAPDH F: 5′-GTCTCCTCTGACTTCAACAGCG-3′; R: 5′-ACCACCCTGTTGCTGTAGCCAA-3′ was used as the endogenous control. Primers were reconstituted in nuclease-free H_2_O for a 100 μM concentration. Primers were diluted to 10 μM for qRT-PCR protocol.

The quantitative reaction was completed using qPCRBIO SyGreen 1-Step Go Lo-ROX (Genesee Scientific, El Cajon, CA, USA; cat no. 17-602B) according to manufacturer’s protocol. The RT-PCR was run using a QuantStudio 5 Real-Time PCR Instrument (96-Well) 0.2 mL Block (Applied Biosystems by ThermoFisher, Waltham, MA, USA; reference no. A28134) with a denaturation step for 10 min at 45 °C–55 °C, annealing step for 2 min at 95 °C, and a final extension step for 4 s at 95 °C and 20 s at 60 °C–65 °C, repeated 40 times. QuantStudio Design & Analysis software was used for determining the relative quantification (RQ) of the gene of interest.

### 2.4. CRISPR-Interference

CRISPRi methodology was used for selective transcriptional repression of DUXAP10 using the protocol described below.

### 2.5. Preparation of Selection Media and Selection Plates

Luria Broth with 100 μg/mL ampicillin was used as selection media and prepared with 10 g/L NaCl, 10 g/L tryptone, and 5 g/L yeast extract. Luria Broth selection plates were made using 12.5 g of Luria Broth mixture combined with 10 g of agar in 1 L of sterile H_2_O with 100 μg/mL ampicillin.

### 2.6. Single Guide RNA Selection

Single guide RNA (sgRNA) sequences were generated using CRISPick software provided by the Broad Institute [13,14] with a Human GRCh38 reference genome and SpyoCas9 as the enzyme (PAM policy, NGG). The top two ranked sgRNA sequences were selected and restriction enzyme overhangs were added for recombination (GeneWiz). Oligonucleotide sequences used:

DUXAP10_sg1 (F) 5′-CACCGGGCGTGGTCAGAGCGAGCTT-3′; (R) 5′-AAAACAAGCTCGCTCTGACCACGCC-3′; DUXAP10_sg2 (F) 5′-CACCGGAGCTTCGGAGAAGCAGTGG-3′; (R) 5′-AAACCCACTGCTTCTCCGAAGCTCCGGTG-3′; 

Non-specific (NS)_sg (F) 5′-CACCAATCTCGCTTATATAACGAG-3′; (R) 5′-AAACCTCGTTATATAAGCGAGATT-3′.

A stock and working solution of 100 μM using 1X TE solution was prepared upon sgRNA arrival for both forward (F) and reverse (R) oligonucleotides.

### 2.7. Single Guide RNA Annealing

The sgRNAs were annealed in a reaction composed of 100 μM F and of 100 μM R sgRNAs, T4 Ligase Buffer, and Polynucleotide Kinase (PNK), quantum satis molecular grade water for a 40 μL reaction. The annealing mixture was incubated at 37 °C for 60 min followed by 95 °C for 5 min, decreasing by 1 °C each minute until a temperature of 12 °C was reached.

### 2.8. Restriction Enzyme Plasmid Digestion

The pLV hU6-sgRNA hUbC-dCas9-KRAB-T2a-Puro (Addgene #71236) used for recombination was gifted from the Charles Gersbach laboratory. The restriction enzyme digestion reaction was made per μg of p71236 plasmid DNA and contained 5 μL of 10X NEB Buffer 3.1, 1 μL of BsmbI Restriction Enzyme, quantum satis molecular grade water for a 50 μL reaction. The digestion mixture incubated at 55 °C for 30 min. Calf intestinal phosphatase (CIP) was added for the remaining 30 min at 37 °C. The reaction mixture was PCR purified prior use for ligation reaction. All 10 μL (500 ng) of PCR purified BsmbI-digested CIP-treated dCas9-KRAB-Puro was used for ligation.

### 2.9. Plasmid Ligation and Recombination

The entirety of the PCR purified digested plasmid was combined with previously annealed sgRNA oligonucleotide mixture with T4 Ligase Buffer and T4 Ligase and incubated at room temperature for 2 h.

### 2.10. Bacterial Transformation of E. coli

NEB-5 alpha *E. coli* was used for transformation and subsequent replication of plasmid DNA. *E. coli* cells were transformed with a 30 s heat shock at 42 °C followed immediately with ice. SOC media was added for recovery and newly transformed bacteria were incubated at 37 °C and 250 rpm. Bacteria were plated on prepared ampicillin selection plates and incubated overnight at 37 °C. Individual colonies were selected and grown in Luria Broth overnight at 37 °C at 250 rpm. Bacterial plasmids were isolated as previously described [15,16]. Recombinant plasmids were sequenced using a primer for the U6 promoter (5′-GACTATCATATGCTTACCGT-3′).

### 2.11. Lentivirus Preparation

HEK293T cells were used for lentiviral harvesting. Components for lentiviral assembly included a pCMV-VSV-G envelope glycoprotein vector (Addgene, Watertown, MA, USA; #12259), psPAX2 (Addgene, Watertown, MA, USA; #12260) packaging vector, and recombined pLV hU6-sgRNA hUbC-dCas9-KRAB-T2a-Puro with gene target. Lentiviral packaging was accomplished using assembly plasmids and a solution of OptiMEM (ThermoFisher, Waltham, MA, USA; cat no. 31985062) and TransIT LT1 Reagent (Millipore, Burlington, MA, USA; cat no. MIR2304).

### 2.12. Lentivirus Transduction

Filtered lentiviral complexes were transduced into target cells plated at 30% confluence and completed using Polybrene Transfection Reagent (10 mg/mL) (Millipore, Burlington, MA, USA; cat no. TR-1003-G) diluted 1:2.5 in 1X sterile PBS and then diluted again 1:1000 in complete media. Cells were incubated at 5% CO_2_ and 37 °C for 48 h.

### 2.13. Puromycin Selection

Concentration of puromycin needed for successful target cell selection following transduction was previously standardized. Cell media was replaced with 8 μg/mL puromycin diluted into complete media for T238. Cells were incubated for 48–72 h at 5% CO_2_ and 37 °C for full selection process. Selected cells were used for all further experimentation.

### 2.14. RNA-Seq

Total isolated RNA from T238-NS and T238-ΔDUXAP10#2 was prepared, in triplicate, as described using Zymo Research Quick RNA Miniprep Kit (cat no. 11237) according to manufacturer’s protocol. RNA was sent frozen for Next Generation Sequencing analysis provided by Azenta, Burlington, MA, USA. Top DEGs and corresponding gene ontology analysis was completed and used for further experimental evaluation.

### 2.15. Click-IT RNA Imaging Assay

The Click-iT RNA Imaging assay was completed according to protocol (ThermoFisher, Waltham, MA, USA; cat nos. C10329, C10330) and was used for nascent RNA labeling. T238 cells were seeded into 6-well plates at a density of 50,000 cells per well to achieve 40–50% confluence in 24 h. Cells were imaged using a Nikon Eclipse TS100 Inverted Microscope green fluorescence channel. Captured cells containing newly synthesized, labeled RNA were quantified using ImageJ. Complete Total Cell Fluorescence (CTCF) was calculated using cell fluorescence measures with subtracted background.

### 2.16. RNA-FISH

Cells were seeded in a 24-well tissue culture treated plate at densities required for 30–40% confluence. Fluorescent labeling of DUXAP10 was completed using QuantiGene ViewRNA ISH Cell Assay according to protocol (cat no. QVC001) and ThermoFisher compatible custom probe sets (cat no. VA4-310-2900). A working probe set solution was prepared at a 1:100 ratio and cells were incubated for 5 hr at in a dry incubator set to 40 °C following lysis with Triton X-100. Cells were imaged using the green fluorescent channel (Alexa Fluor^TM^ 488) using a Morrel Nikon Eclipse TS100 Inverted Microscope.

### 2.17. Colony Forming Unit Assay

Colony forming units (CFU) were used to evaluate clonogenic capacity. Cells were plated at sparse even densities (200 cells/well) in a 6-well tissue culture treated plate. Cells were incubated at 37 °C and 5% CO_2_ and monitored for colony formation. Endpoint was reached when wells containing control T238 had maximum colony formation. Cells were fixed using 100% methanol and stained using 1% toluidine blue in 1% borax solution.

### 2.18. XTT Assay

Non-radioactive cell viability was quantified over 72 h using a sodium 3′-[1-(phenylaminocarbonyl)-3,4-tetrazolium]-bis (4-methoxy6-nitro) benzene sulfonic acid hydrate (XTT) substrate. Colorimetric XTT assay was completed according to manufacturer’s instructions (Millipore Sigma, St. Louis, MO, USA; product no. 11465015001). 1 mg of XTT with 2.5 μL phenazine methosulfate (PMS) (Sigma, St. Louis, MO, USA; cat no. P58-12) was added to clear 1X RPMI. Incubation time was previously evaluated for each cell line and was determined to be 6 h at 37 °C and 5% CO_2_ for T238. Absorbances were measured with wavelengths set to 450 nm/640 nm.

### 2.19. Cell Death Detection ELISA

Apoptotic induction in response to starvation of cells was evaluated and measured using a Cell Death Detection ELISA (Sigma, St. Louis, MO, USA cat no. 11544675001) according to manufacturer’s instructions. Samples for analysis were prepared using adherent cells in a 96-well tissue culture treated plate at a density of 1000 cells in serum-free media. Cells were incubated for 4 h at 37 °C and 5% CO_2_.

### 2.20. Invasion Assay

Invasion Index was evaluated using Corning Chambers coated with a Matrigel Matrix according to manufacturer’s instructions (Corning, Corning, NY, USA; cat no. 354483). Approximately 10,000 cells were seeded into each coated insert containing serum-free media. Complete media with chemoattractant was placed in the wells. Cells were incubated for 18 h at 37 °C and 5% CO_2_. Invaded cells were stained with 1% toluidine blue in 1% borax solution for 2 min and placed in distilled H_2_O for 2 min for rinsing. Invaded cells were then imaged using Bright Field Microscopy at 40X. Cell counts were normalized to number of cells migrated to determine Invasion Index.

### 2.21. Migration Assay

Migration was evaluated using Corning Falcon Multiwell Insert System control inserts according to manufacturer’s instructions (cat no. 351185). Approximately 10,000 cells were seeded into each insert containing serum-free media. Complete media with chemoattractant was placed in the wells. Cells were incubated for 18 h at 37 °C and 5% CO_2_. Migrated cells were stained with 1% toluidine blue in 1% borax solution for 2 min and placed in distilled H_2_O for 2 min for rinsing. Migrated cells were then imaged using Bright Field Microscopy at 40X.

### 2.22. Scratch Wound Assay

Cells were seeded into 24-well tissue culture treated plates at a density of 150,000 cells per well. Cells were incubated for 24 h at 37 °C and 5% CO_2_ and checked to ensure 100% confluence. A sterile 20 μL pipette tip was used to introduce a wound field directly through the center of the well. The wound field was measured using Bright Field Microscopy. Cells were incubated for 18–20 h at 37 °C and 5% CO_2_. Wound field measurements were recorded, and calculations were made according to the following equation:

Percent Healed (%) = (Wound Field Measurement at T = 22/Wound Field Measurement at T = 0) × 100%

### 2.23. Tumorigenesis Mouse Model

Cells were grown and harvested using complete media. Approximately 500,000 cells were resuspended in sterile 1X PBS and kept on ice. Using a 24-gauge needle and 1 cc syringe, approximately 200 μL of cell suspension was administered into the hind leg flank of Female NU/J Mice Strain 002019 (Jackson Laboratory). Palpable tumors present after 3 weeks were measured using a vernier caliper and recorded for volume calculations. At the termination of the experiment (tumors > 2 cm), tumors were removed and weighed for evaluation of tumorigenesis. Gross lung images were taken and using ImageJ v2 software, were used to calculate the Metastatic Area using the following formula:

Metastatic Area (%) = (Tumor area/Total area) × 100%

All in vivo experiments were conducted in the Department of Comparative Medicine at New York Medical College under the approved Institutional Animal Care and Use Committee (IACUC) protocol number 23755.

## 3. Results

### 3.1. Bioinformatic Analysis

**Figure 1:**
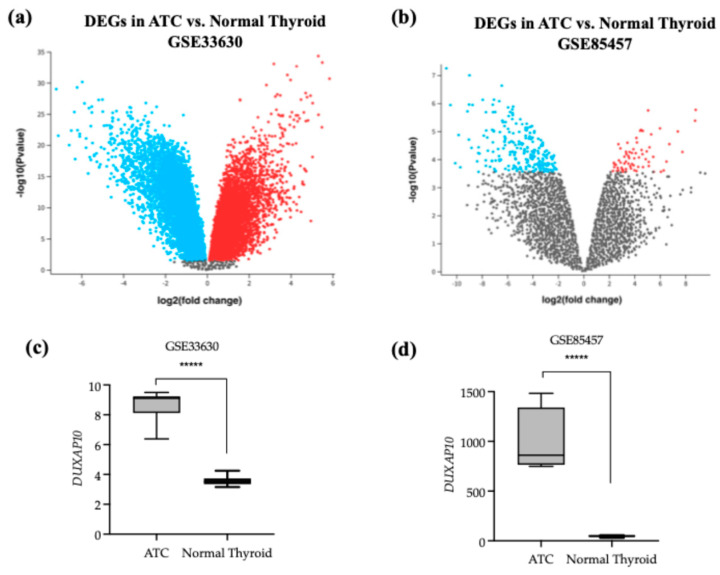
DUXAP10 is highly expressed in ATC compared with normal thyroid tissue. (**a**,**b**) GSE33630 dataset showing the top 250 differentially expressed genes (DEGs) and relative DUXAP10 expression in ATC and normal thyroid tissue samples; Red represents genes upregulated in ATC vs. normal and blue represents genes downregulated in ATC vs. normal; (**c**,**d**) GSE85457 dataset showing the top 250 DEGs and DUXAP10 expression in ATC and normal thyroid primary cells. Statistical analysis: Wald test in GEO2R (Limma package, R). ***** *p* ≤ 0.0000001.

**Figure 2:**
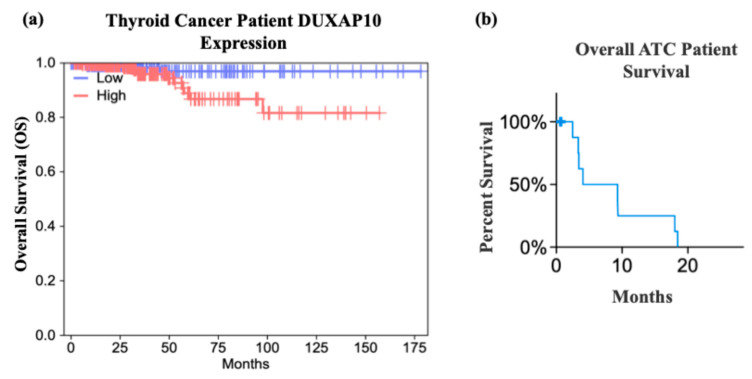
GEPIA and cBioPortal analyses of DUXAP10 expression in ATC patients. (**a**) Kaplan–Meier survival analysis from TCGA-THCA data (GEPIA); (**b**) cBioPortal datasets of percent survival of ATC patients with high DUXAP10 expression. Statistical analysis: Log-rank test (95% CI).

To identify dysregulated lncRNAs in ATC, we analyzed publicly available NCBI GEO datasets using the GEO2R tool to determine the top 250 DEGs from the Affymetrix Human Genome U133 Plus 2.0 Array (Figure 1a,c). DUXAP10 was significantly overexpressed in ATC compared to normal thyroid tissue in both GSE33630 and GSE85457, with increases of 40-fold and 20-fold, respectively (Figure 1b,d). Because differential expression alone does not imply functional relevance, we used GEPIA to evaluate clinical correlations. High DUXAP10 expression was significantly associated with decreased overall survival in thyroid cancer patients (Figure 2a). Further analysis of ATC patient genomic data provided by cBioPortal, which included 213 ATC and 115 PTC samples [17], showed that ATC patients with high DUXAP10 expression had lower survival (Figure 2b).

### 3.2. Generation of In Vitro Cell Model Using CRISPR-Interference (CRISPRi)

**Figure 3:**
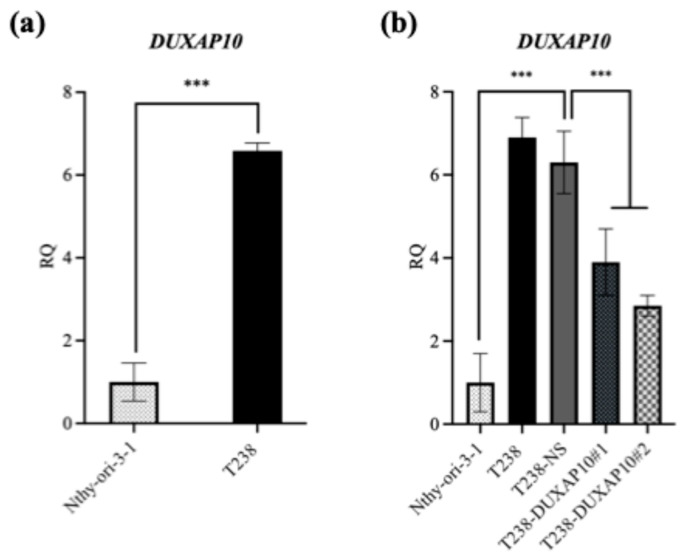
DUXAP10 expression is significantly overexpressed in ATC cell line T238 compared to in immortalized normal thyroid follicular epithelial cell line Nthy-ori3-1. (**a**) DUXAP10 expression in ATC cell line T238 and normal thyroid epithelial cell line Nthy-ori 3-1; (**b**) DUXAP10 expression was reduced by 40% and 50% in T238-ΔDUXAP10#1 and T238-ΔDUXAP10#2 CRISPRi cell lines, respectively. Statistical analysis: Mann–Whitney non-parametric *t*-test; *n* = 6 per group; *** *p* ≤ 0.001.

**Figure 4:**
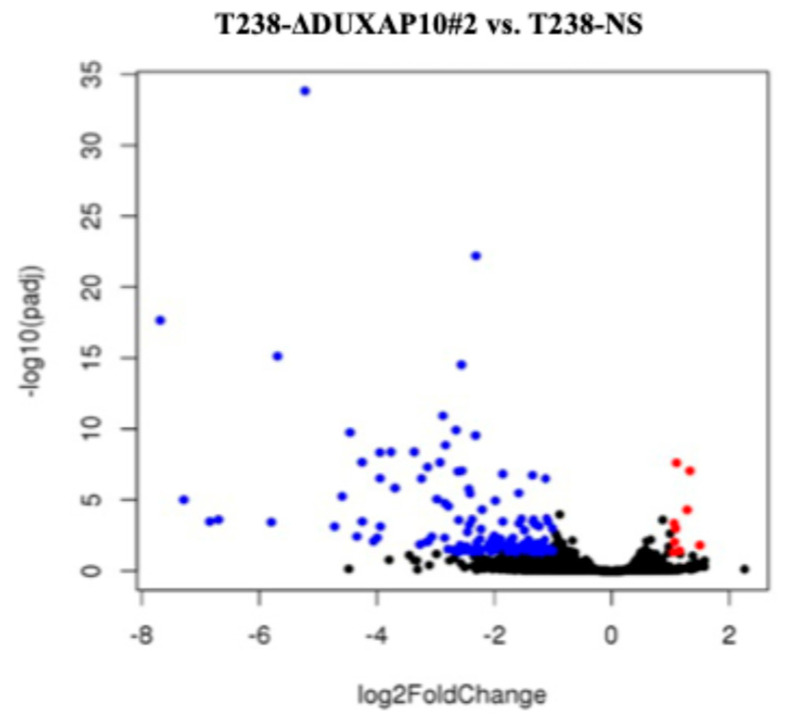
RNA-seq analysis reveals transcriptional repression of DUXAP10 in T238 results in extensive gene expression changes. Volcano plot of DEGs in T238-NS vs. T238-ΔDUXAP10#2 knockdown. Red represents genes that are upregulated in T238-ΔDUXAP10#2 vs. T238-NS and blue represents genes that are downregulated in T238-ΔDUXAP10#2. Black represents non-significant DEGs. Statistical analysis: DESeq2 software (Azenta) with Wald test.

To investigate DUXAP10 function, we generated ATC cell lines with transcriptionally repressed DUXAP10 expression using CRISPRi. Using qRT-PCR, we first observed that DUXAP10 expression is significantly upregulated in ATC cell line T238 compared to wild-type thyroid epithelial cell line Nthy-ori-3-1 (Figure 3a). Using T238, we generated two DUXAP10 CRISPRi knockdown cell lines (T238-ΔDUXAP10#1, T238-ΔDUXAP10#2), which were evaluated alongside our scrambled vector CRISPRi control (T238-NS). CRISPRi-mediated repression decreased DUXAP10 expression by 40% in T238-ΔDUXAP10#1 cells and by 50% in T238-ΔDUXAP10#2 cells (Figure 3b). RNA-seq analysis revealed over 120 DEGs in the T238-ΔDUXAP10#2 knockdown compared to T238-NS (Figure 4). The majority of these DEGs have been reported to promote cancer processes (proliferation, metastasis, apoptotic evasion, clonogenic capacity, stress resistance, etc.). Thus, the transcriptional repression of a single gene significantly impacted the landscape of gene expression in T238, warranting extensive molecular evaluation of DUXAP10 in ATC to determine how these processes are modified.

### 3.3. DUXAP10 Localizes in the Nucleus of ATC Cell Line T238 and Impacts RNA Output

**Figure 5:**
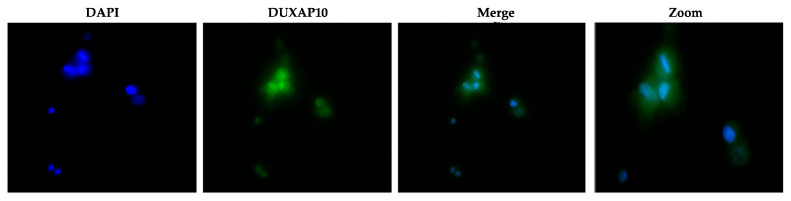
DUXAP10 localizes in the nucleus and cytoplasm of T238. Fluorescent images of RNA-FISH acquired with a Morrel Nikon Eclipse TS100 inverted microscope.

**Figure 6:**
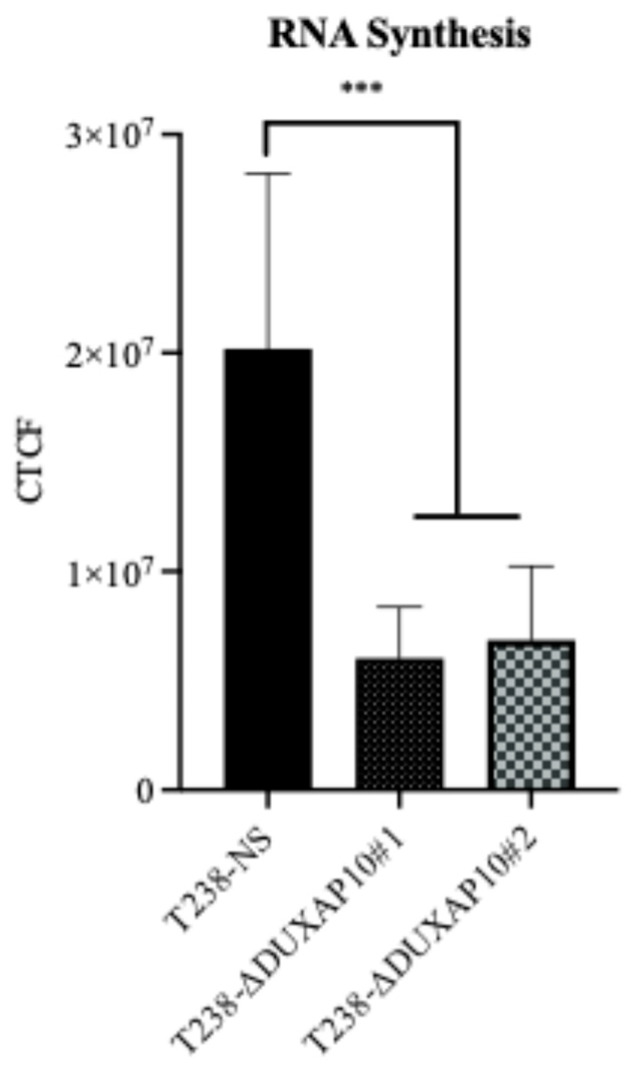
Transcriptional repression of DUXAP10 reduced RNA synthesis of ATC cell line T238. Fluorescent labeling of nascent RNA demonstrated an overall 65% reduction in global RNA synthesis in T238-ΔDUXAP10 knockdowns. Images acquired using the green fluorescent channel (Alexa Fluor 488) on a Morrel Nikon Eclipse TS100 inverted microscope. Statistical analysis: Mann–Whitney non-parametric *t*-test; *n* = 9 per group; *** *p* ≤ 0.001.

The subcellular localization of lncRNAs strongly influences their mechanism of action. Using RNA-FISH, we determined that DUXAP10 is located primarily in the nucleus of T238, with some cytoplasmic presence (Figure 5). This suggests that DUXAP10 may regulate key cellular processes through its DNA-, RNA-, and protein-binding interactions. To gain a sense of DUXAP10’s overall impact on the cellular output of T238, we evaluated the rate of RNA synthesis. RNA output of cancer cells is extensively accelerated, which contributes to the increased needs of cancer cells and their immortalization. Using a “ClickRNA” reaction, we labeled nascent RNA during synthesis using 5-EU conjugated with a fluorescently tagged azide molecule. We determined that the transcriptional repression of DUXAP10 reduced the rate of T238’s RNA synthesis, quantified by the by 65% decrease in fluorescent labeling (Figure 6).

### 3.4. CRISPRi-DUXAP10 Cell Model Significant Reduces Cell Sustainability Phenotypes in Vitro and in Vivo

**Figure 7:**
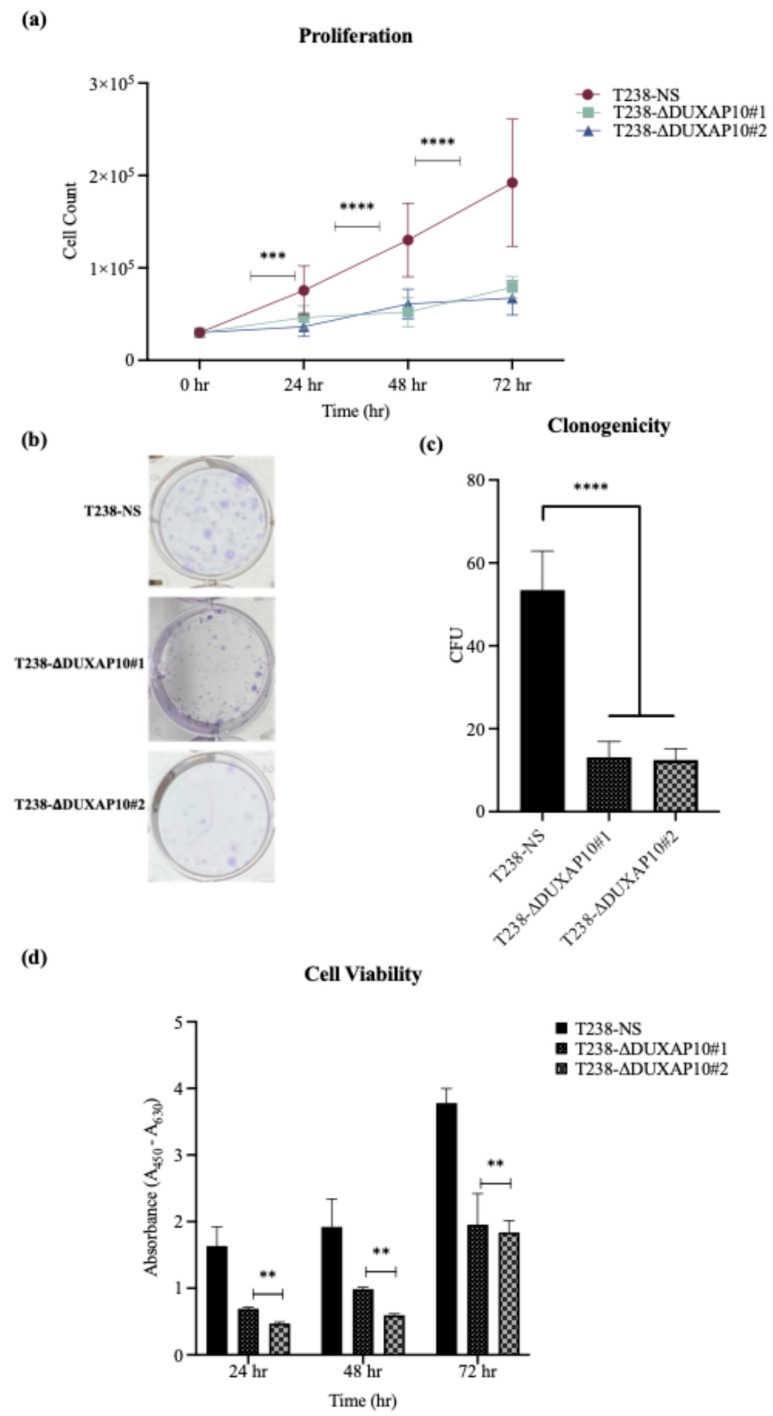
Transcriptional repression of DUXAP10 reduced growth and clonogenic potential of ATC cell line T238. (**a**) Quantification of cell counts over 72 h; (**b**) Images of stained CFUs; (**c**) Quantification of CFUs after 14 days; (**d**) XTT absorbance values over 72 h. Statistical analysis: Mann–Whitney non-parametric *t*-test; *n* = 9 per group; ** *p* ≤ 0.01; *** *p* ≤ 0.001; **** *p* ≤ 0.0001.

**Figure 8:**
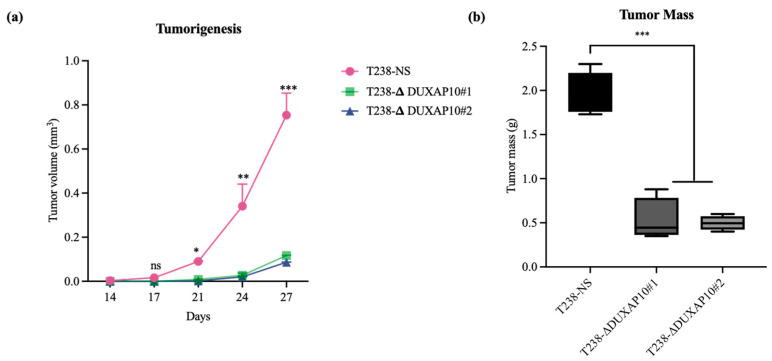
Transcriptional repression of DUXAP10 reduced ATC tumorigenesis in vivo. (**a**) Tumor volume measurements over 36 days; (**b**) Post-mortem tumor mass at day 36. Statistical analysis: Mann–Whitney non-parametric *t*-test; *n* = 5; * *p* ≤ 0.05; ** *p* ≤ 0.01; *** *p* ≤ 0.001; ns = not significant.

The significant reduction in RNA output led to our investigation of how DUXAP10 expression impacts phenotypes associated with cell sustainability. We first observed, using a Trypan Blue Cell Exclusion Assay, that the transcriptional repression of DUXAP10 reduced the proliferative capacity of T238 significantly, over the course of 72 h (Figure 7a). Given the marked reduction in proliferation observed in T238-ΔDUXAP10 knockdown cells, we next evaluated their capacity for self-sufficiency. Using a CFU assay, we determined that the transcriptional repression of DUXAP10 reduced the clonogenic capacity of T238 by 70% (Figure 7b,c). This result was supported by a significant reduction in cell viability over the course of 72 h, which was evaluated by the quantification of XTT reduction (Figure 7d). Subcutaneous implantation of our T238 control and knockdown cell lines in athymic nude mice revealed a significant reduction in tumorigenesis (Figure 8). This was confirmed by both tumor volume (Figure 8a) and weight (Figure 8b).

**Figure 9:**
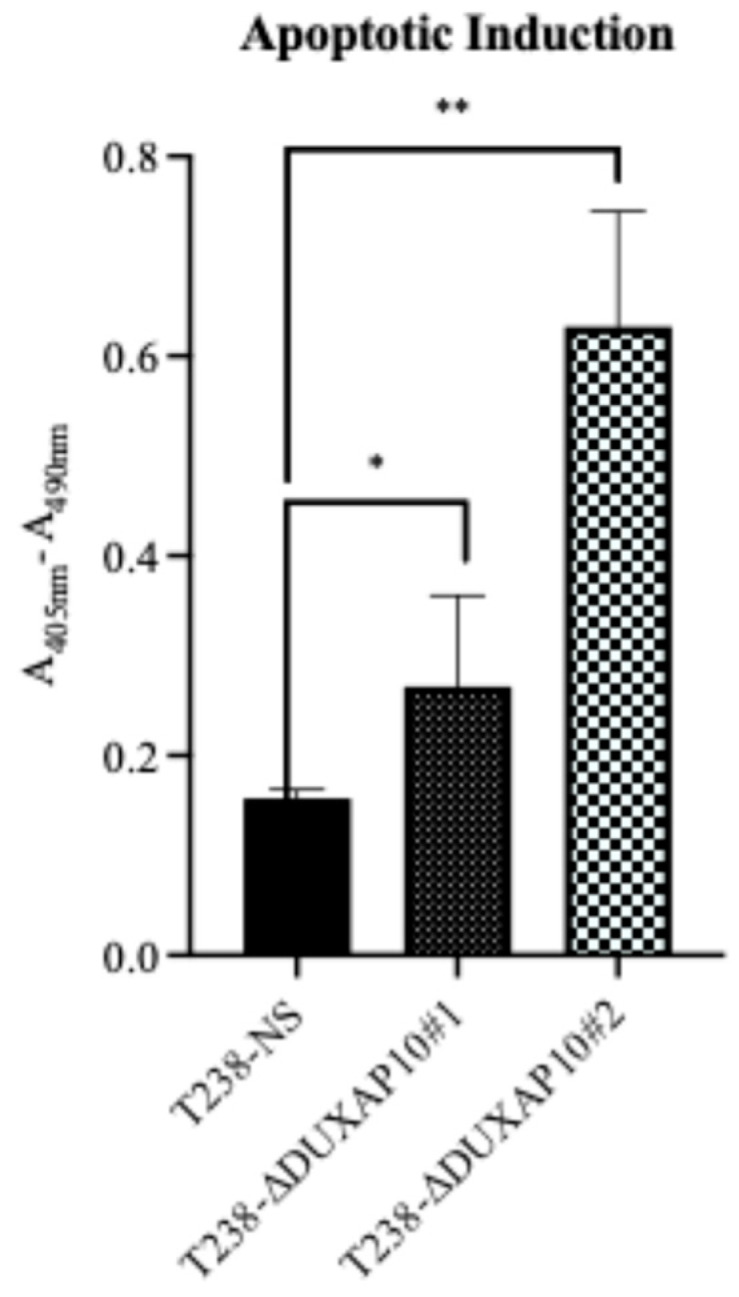
Transcriptional repression of DUXAP10 increased apoptotic induction in ATC cell line T238. Apoptotic activity quantified in T238-ΔDUXAP10 knockdown lines showed significantly higher induction compared to the T238 control. Statistical analysis: Mann–Whitney non-parametric *t*-test; *n* = 6; * *p* ≤ 0.05; ** *p* ≤ 0.01.

In addition to a significant impact on the proliferative capacity of T238, the transcriptional repression of DUXAP10 remarkably increased the induction of apoptosis in response to nutrient deprivation (Figure 9), which was determined using a Cell Death ELISA for histone fragment segmentation.

### 3.5. CRISPRi-DUXAP10 Cell Model Significant Reduces Metastatic Phenotypes In Vitro and In Vivo

**Figure 10:**
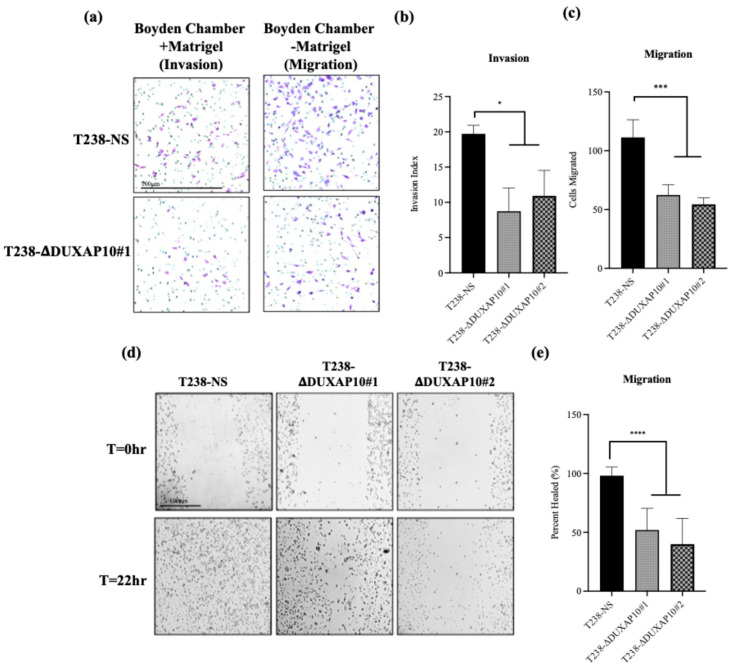
Transcriptional repression of DUXAP10 reduced metastatic phenotypes of ATC in vitro and in vivo. (**a**) Reduced extracellular matrix degradation and migration in Boyden-chamber assays; (**b**,**c**) Quantification of ‘Invasion Index’ and migratory cells; (**d**,**e**) Scratch-wound healing assay. Statistical analysis: Mann–Whitney non-parametric *t*-test; *n* = 10; * *p* <0.05, *** *p* < 0.001, **** *p* < 0.0001.

**Figure 11:**
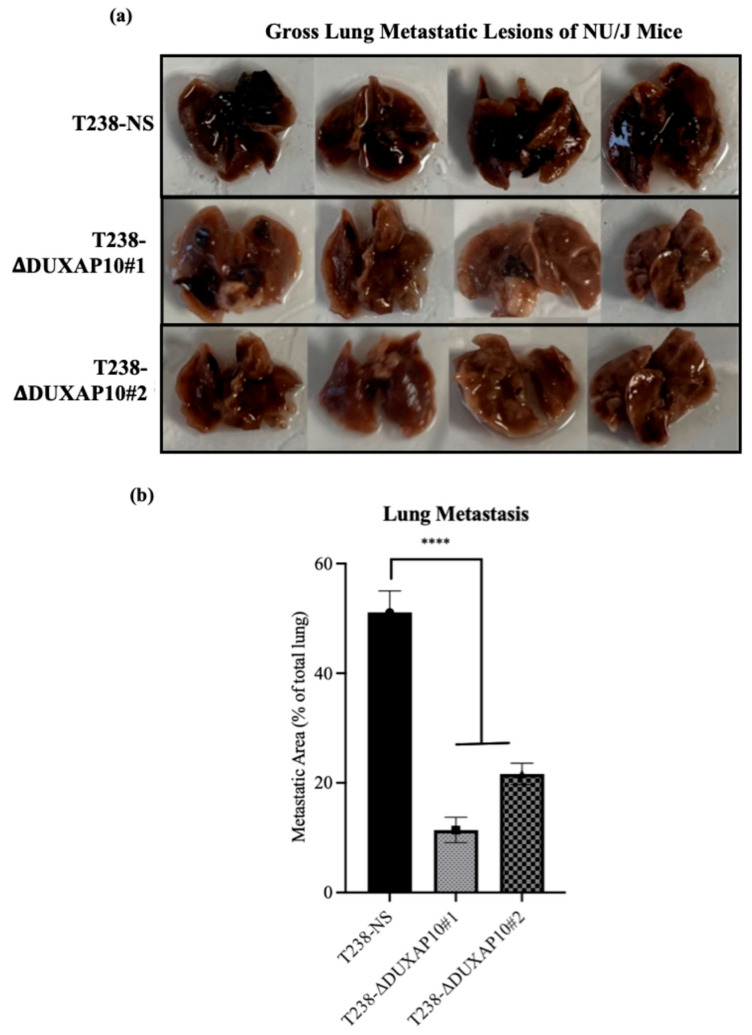
Transcriptional repression of DUXAP10 reduced lung metastatic burden of ATC in vivo. (**a**) Representative images of metastatic lung lesions extracted from athymic mice post-mortem; (**b**) Image-based (ImageJ) quantification of lung metastatic area. Statistical analysis: Mann–Whitney non-parametric *t*-test; *n* = 4; **** *p* ≤ 0.0001.

To determine DUXAP10’s impact on phenotypes associated with metastasis in vitro, we used a Boyden Chamber Assay to evaluate invasion and migration. We observed that the transcriptional repression of DUXAP10 decreased the invasion index of T238 by 40%, as well as a 50% decrease in migratory capacity (Figure 10a–d). Using a scratch wound assay, we also confirmed a 50% reduction in migration in both T238-ΔDUXAP10 knockdown cell lines (Figure 10e,f). A substantial decrease in lung metastatic lesions was also observed in our athymic nude mice that had tumors with T238-ΔDUXAP10#1 and T238-ΔDUXAP10#2 implanted, confirmed by a 70% reduction in metastatic area (Figure 11). These findings confirm that DUXAP10 repression reduces metastatic behavior both in vitro and in vivo.

## 4. Discussion

Cancer genomics is a complex and evolving field of research. Studying uncharacterized molecular patterns has led to the discovery of novel biomarkers and therapeutic targets. Furthermore, researchers have begun to describe cancer types that exhibit unique biological puzzles. The inherent metastatic landscape of ATC presents significant diagnostic, prognostic, and therapeutic hurdles. Although mutational burden contributes to ATC pathology, it provides limited insight for diagnosis or treatment. Defining the genomic and transcriptomic landscape of ATC, as well as PTC and follicular thyroid cancer (FTC) tumors, can help improve early detection, treatment regimen, and ultimately, patient outcomes [17,18,19]. In support of our research findings, it is reported that gene expression analysis can help curate molecular signatures that can determine tumor staging and therapeutic responsiveness—revolutionizing the standard of care in the field of oncology [20,21,22,23]. Moreover, the novelty and success of transcriptomic investigation, specifically the non-coding portion, cannot be overlooked [24].

The limited success of using small-molecule inhibitors in ATC, as well as the inability to surgically resect or treat metastatic ATC, further highlights the need for additional avenues of intervention. This study aimed to identify a novel dysregulated gene that can serve as either a prognostic, diagnostic, or therapeutic target in ATC. Advances in sequencing and biomarker discovery serve as promising avenues for combating ATC [25,26]. Molecular signatures that orchestrate ATC’s unique pathology will significantly aid in disease management and curative efforts. Investigation of a more novel set of cellular regulators, such as lncRNAs, will help decipher the ways in which ATC is initiated and further driven— exceeding the bounds of mutational study. This pipeline of research has been successfully applied to many other cancer types. Publicly available datasets provided by NCBI were analyzed and indicated that DUXAP10 is a significantly overexpressed transcript and was the top statistically significant DEG in dataset GSE33630 (Figure 1). The study provided by cBioPortal contained genomic datasets of well-differentiated thyroid cancer patients and found that the initial mutational drivers have striking similarity (i.e., mutations in MAPK and RAS); however, ATC acquires a large mutational burden as it progresses. This concept was also exhibited in ATC with concomitant well-differentiated thyroid cancer (i.e., PTC). It was reported that ATC and well-differentiated thyroid cancer share common ancestral cells and diverge as disease progresses [27]. These data support our hypothesis that DUXAP10 expression may serve as a prognostic marker in early ATC, and/or its upregulation heavily impacts the transition of patients into a stage IVC metastatic diagnosis, and GEPIA confirms a significant impact on patient survival when overexpressed (Figure 2). In support of the initial selection of DUXAP10, this molecule is derived from homeobox genes, which are indispensable molecular elements during embryonic development. It has been discovered in recent years that homeobox genes, encoding for distinct homeobox proteins, serve as oncogenes or tumor suppressors in nearly all cancer types [28]. Thus, a homeobox pseudogene-derived lncRNA, such as DUXAP10, is an extremely attractive molecule for research and intervention.

To understand the consequences of DUXAP10 overexpression in ATC, we employed CRISPRi technology to transcriptionally repress DUXAP10 at the genomic level—targeting two distinct regions of its promoter. The generation of stable CRISPRi cell lines facilitated comprehensive functional analyses of DUXAP10 and addressed the inherent limitations of transient RNA interference methodologies (Figure 3). By result of DUXAP10 transcriptional repression in ATC cell line T238, a substantial impact of gene expression was observed using RNA-seq analysis (Figure 4). A majority of these DEGs encode for proteins that are known to play pivotal, or minor roles across many cancer types. LncRNAs are functionally classified as miRNA sponges, protein scaffolds, and signal decoys. Therefore, studying DUXAP10, a large lncRNA molecule, is advantageous and likely has multi-faceted biological capabilities, which explains the drastic alteration of gene expression shown in Figure 4. We observed that DUXAP10 is expressed in both the nucleus and cytoplasm of ATC cells, with a strong nuclear localization. This suggests that DUXAP10, functionally, can regulate DNA and RNA expression, as well as serve as a protein-binding molecule (Figure 5). A critical, functional role for DUXAP10 was confirmed again by the marked reduction in RNA output, a key component of cell survival. These results provided evidence that DUXAP10 expression does significantly impact the S-phase of the cell cycle (Figure 6). The rate at which RNA is synthesized is drastically increased in cancer due to the dysregulated expression of transcription factors and RNA polymerases [29]. Previous studies have also shown the significant impact that targeting RNA polymerase II as a therapeutic option in some cancers— further supporting our finding that DUXAP10 expression alters this output [30]. Data shown in Figure 6 supported the RNA-seq data, which revealed the impact of DUXAP10 expression on the output of over 100 cancer-promoting genes. Published work on these genes has described them as bona fide regulators of proliferation, apoptosis, invasion, migration, etc. encompassing a broad spectrum of cancer-associated phenotypes. Therefore, we wanted to investigate if there is a phenotypic translation of this data.

Ultimately, we described a significant role for DUXAP10 in phenotypes associated with ATC cell sustainability and tumorigenesis (Figure 7 and Figure 8). Hence, the reduction in proliferation, clonogenic capacity, and cell viability in both T238-ΔDUXAP10 knockdown cell lines supported the previously observed alteration in global transcription, as well as gene-specific regulation (Figure 4 and Figure 6). The balance of immortality of cancer cells lies in the upregulation of proliferative pathways, followed by the loss of negative regulation that is controlled by apoptotic induction. LncRNA molecules can drive proliferative signaling while promoting the loss of cell-death signals. Our data showed that in response to starvation, our T238-ΔDUXAP10 knockdown cell lines had significantly increased the sensitivity and activation of apoptosis, which was marked by the presence of mono-and oligo-nucleosome production— key markers of cell death (Figure 9).

A key component of ATC is its inherent metastatic propensity. The activation of Epithelial-to-Mesenchymal Transition (EMT) processes aids in metastasis of cancer cells to distant secondary, tertiary, etc., sites [31]. In ATC, metastasis occurs rapidly, typically in the weeks that follow disease propagation. The rapid proliferation at the ATC tumor sites contributes to the loss of nutrient supply and inherent need for evacuation and re-establishment at distant anatomical sites [32]. We observed that both T238-ΔDUXAP10 knockdown cell lines significantly reduced the migratory and invasive capabilities of ATC in vitro and in vivo. EMT is supported by the loss of a stagnant, epithelial-like phenotype, followed by the upregulation of a motile, mesenchymal-like phenotype. We observed, using a pseudo-extracellular Matrigel matrix, that the T238-ΔDUXAP10 knockdowns had a reduced ability to degrade the components that make up a physiological extracellular matrix (Figure 10a,b). This was supported by the significant reduction in migratory capacity, measured by both the reduction in movement towards a chemoattractant (Figure 10a,c) and ability to seal a wound field (Figure 10d,e). When implanted in our athymic mouse model, the significant reduction in tumor growth (Figure 8) was supported by a reduction in metastatic lung lesions observed post-mortem (Figure 11). Thus, our results heavily support a simultaneous role for DUXAP10 in the orchestration of ATC cell growth, cell death, and metastatic capabilities. We propose that DUXAP10 employs its protein- and nucleic acid-binding domains to modulate gene expression. Whether this occurs through direct interaction with chromatin, transcription factors, or other regulatory complexes remains an important avenue for future investigation. Further research regarding the molecular interactions of DUXAP10 that regulate these functions is warranted.

## 5. Conclusions

This research presents one example of how a singular lncRNA molecule can utilize its multi-ranged capabilities to facilitate nearly all processes of carcinogenesis and metastasis. The transcriptional repression of DUXAP10, a single lncRNA, had a substantial impact on the entire molecular and phenotypic profile of ATC both in vitro and in vivo. The homeobox-derived nature of this molecule underscores its developmental potency and potential oncogenic reactivation in malignant settings. Given its minimal expression in normal tissue but striking overexpression in ATC, DUXAP10 represents a compelling prognostic marker and therapeutic target. The strategic upregulation of this transcript across several cancer types underscores the nuanced regulation of its expression. Future work should describe its molecular interactors and assess the feasibility of lncRNA-directed therapeutics in aggressive thyroid cancers. The use of CRISPRi highlights this concept, as complete knockout and loss of expression may result in unwanted and inadvertent impacts on the result and analysis. The recapitulation of patient genomic profiling through the use of our in vitro and in vivo cell and murine models enabled the conclusion that DUXAP10 plays a critical role in the tumorigenic and metastatic landscape of ATC. Future studies should aim to define DUXAP10’s complete interactome and assess whether targeted silencing could mitigate ATC progression in preclinical models.

## Data Availability

The original contributions presented in this study are included in the article. Further inquiries can be directed to the corresponding authors.

## Abbreviations

The following abbreviations are used in this manuscript:

ATC	Anaplastic Thyroid Cancer
PTC	Papillary Thyroid Cancer
DEGs	Differentially expressed genes
LncRNA	Long non-coding RNA
NCBI	National Center for Biotechnology Information
GEO	Gene Expression Omnibus
DUXAP10	Double Homeobox A Pseudogene 10
TPM	Transcripts per million
RQ	Relative quantification
CRISPRi	CRISPR-interference
sgRNA	single guide RNA
PNK	Polynucleotide Kinase
CIP	Calf intestinal phosphatase
CTCF	Complete Total Cell Fluorescence
CFU	Colony forming units
IACUC	Institutional Animal Care and Use Committee
GEPIA	Gene Expression Profiling Interactive Analysis
OS	Overall Survival
TCGA	The Cancer Genomic Atlas
THCA	Thyroid Cancer
